# Life after COVID-19: preparing for changes in mental healthcare service demand

**DOI:** 10.1017/ipm.2020.73

**Published:** 2020-06-02

**Authors:** Owen P. O’Sullivan

**Affiliations:** Fellow in Medical Education, South London and Maudsley NHS Foundation Trust, London, UK; Specialty Registrar, North East London Forensic Psychiatry Training Scheme, (Email: owenosullivan@rcsi.ie)

Dear Editor,

The COVID-19 pandemic and efforts to contain its spread have undoubtedly lead to disruptions in the continuity of mental healthcare. In and of itself, it may also lead to deteriorations in existing mental health conditions in addition to new presentations. Disruptions in care, service reconfigurations, staff shortages and challenges in migrating elements of mental health services to digital platforms – and ensuring equitable access – will all complicate the delivery of safe and effective care during this crisis. In addition to mental health conditions, it has been proposed there will also be increases in levels of loneliness, substance misuse, domestic violence and child abuse (Galea, Merchant & Lurie, 2020).

Whilst critically necessary in terms of protecting public health, restrictive measures are not without their cost in terms of mental health across the population. A very recent rapid review examined the literature in relation to the psychological impact of quarantine (Brooks *et al.* 2020). Across a range of quarantine experiences in various infectious disease contexts in different countries, the authors determined its potential effects to be substantial and potentially long-lasting. They called for its use to be evidence-based and that every measure be made to render it as tolerable as possible for the population. Most studies included in the report referenced damaging psychological effects and cited risk factors, including longer duration, inadequate information, financial loss and stigma. Given the medium- to long-term impact of the crisis at a public health level coupled with its economic consequences, it is reasonable to infer it will lead to increased numbers seeking to access mental healthcare. As such, considering the uncertain future in terms of an easing of the restrictive measures, primary care and mental health services must begin preparations for the downstream effects not just on existing mental health teams’ caseloads, but on the at-risk population also.

However, the potential for psychological damage does not stop there. Essential workers, especially in healthcare, will have been acutely exposed to infectious risk. Many essential non-healthcare workers will also have been exposed to similar risks albeit without their employers having experienced and established infection control protocols to afford them adequate protection. Clinical staff may have been redeployed to unfamiliar settings. On unfamiliar ground, transplanted into unfamiliar teams, they may face the most stressful periods of their careers divorced from their usual support structures, lines of supervision and relative comfort of their own specialty. Many healthcare staff will have potentially traumatic experiences and, indeed, experience personal losses. This will almost certainly be on a background of ongoing contention around issues such as availability of personal protective equipment and implementation and adherence to public health measures. Moreover, so many elements of Ireland’s health services were critically under-resourced and shamefully behind their international counterparts before we ever heard the word ‘coronavirus’.

Mostly studied in war veterans, moral injury refers to profound psychological distress resulting from actions or omissions transgressing deeply held moral beliefs and expectations (Litz *et al.* 2009). Across a range of professions and countries, it has been found to be significantly associated with post-traumatic stress disorder, depression and suicidal ideation (Williamson, Stevelink, & Greenberg, 2018). Clinical staff may be especially vulnerable to moral injury during the current pandemic (Williamson, Murphy, & Greenberg, 2020). Whilst consensus around case definition and rating scales has grown, questions remains about the optimal treatment approach. Jones (2020) summarised the conceptual challenge moral injury presents for psychiatry as follows, ‘clinical interpretation…runs the risk of medicalising ethical behaviour when associated with distress, or pathologising the emotions that arise when a person is presented with a complex or irreconcilable dilemma’. Moral distress and moral injury, whilst not recognised mental health conditions in the traditional sense (i.e. inclusion in the latest versions of the Diagnostic and Statistical Manual of Mental Disorders and International Classification of Diseases: DSM-5 and ICD-11), may transpire to become so as a result of a global crisis on this scale. Indeed, one could argue parallels suggestive of shell shock’s emergence during World War I. Whether or not this crisis will lead to a formal incorporation of moral distress and injury into our current diagnostic classification systems remains to be seen. In any event, healthcare organisations must be alive to the mental health risks this pandemic poses in that regard. Staff deserve nothing less than robust evidence-driven support structures fit-for-purpose to meet their evolving and potentially complex needs.

In terms of shouldering the economic consequences of this pandemic, we will have seen how COVID-19 impacts on professions and sectors in vastly different ways in our own families. Data published in April 2020 show a clear positive association from Irish figures between higher income (based on per capita gross domestic product) and increased likelihood of being able to work remotely (Dingel & Neiman, 2020). This indirectly illustrates how both the public health risk and economic fall-out of the COVID-19 pandemic will likely disproportionately impact on those with lower incomes, at least in the short term. In recent years, many fields of work have seen increasing numbers employed via short-term contracts and through freelancing arrangements. From the early stages of the pandemic, latent vulnerabilities within this ‘gig economy’ have been acutely exposed with the crisis bringing into sharp focus the precarity of many such workers’ employment situations. Many of those unable to work from home will lose jobs. Many others will lose them regardless.

The collective experience of a population in quarantine forces us into stark confrontation with the realities of life for those lacking stable accommodation. However, large-scale mobilisation of state resources to intervene should not be reserved for a crisis such as the present. Ireland’s homelessness crisis predates COVID-19.

In the UK, a governmental inquiry is to be launched to examine emerging evidence that black and minority ethnic (BME) populations are being disproportionately impacted by COVID-19. This has been suggested at a population level and also in strikingly elevated mortality figures among BME health and social care staff (Cook *et al.* 2020). Health inequalities, the underlying social and economic determinants thereof and their interplay with governmental policy will be key areas of research focus in the months and years to come. Ireland must also examine its data and review whether enough is being done to assertively mitigate health inequalities.

The return to normal is neither likely to be swift nor without complication. We can expect a cautious reversal of the restrictive measures coupled with the ever-present spectre of false dawns should pockets of the outbreak re-emerge. Behavioural fatigue, despondency, frustration and even rebellion against the measures may emerge as features of such a phase. Additionally, any reversal of the restrictive measures will be against the backdrop of a global economic system looking to aggressively revive itself. Allied with this, as a population, we will most certainly be subject to an unprecedented marketing onslaught encouraging us to return to normal. It will prove difficult for many to reconcile this with their recent experiences and, in many cases, losses.

Mental health services will have faced many challenges during this recent period in terms of adapting and continuing to support service users and their loved ones. These challenges came rapidly and more will follow. The COVID-19 pandemic also asks of services to prepare to support those who may, in time, come to need mental health services as a result of the direct and in-direct consequences of efforts to contain the spread. In the longer term, this pandemic is likely to further exacerbate existing health and economic inequalities and may also reshape the population’s demand for health and social care in unforeseen ways. To meet these demands, it is imperative that Ireland’s new government gives firm commitment to an unqualified and sustained programme of investment in mental healthcare. Otherwise, services and organisations may struggle to rise to the challenges that await at the other side of COVID-19.
